# Why the Jenner/Pasteur paradigm is insufficient for controlling vector-borne diseases and the role of microbiota-mediated interactions

**DOI:** 10.1016/j.crpvbd.2025.100291

**Published:** 2025-07-08

**Authors:** Ana Laura Cano-Argüelles, Lianet Abuin-Denis, Dasiel Obregon, Lourdes Mateos-Hernandez, Apolline Maître, Elianne Piloto-Sardiñas, Alejandra Wu-Chuang, Pierre Tonnerre, Alejandro Cabezas-Cruz

**Affiliations:** a Parasitology Laboratory, Institute of Natural Resources and Agrobiology of Salamanca (IRNASA, CSIC), Cordel de Merinas, 40-52, 37008, Salamanca, Spain; b Anses, INRAE, Ecole Nationale Vétérinaire d’Alfort, UMR BIPAR, Laboratoire de Santé Animale, Maisons-Alfort, F-94700, France; c Animal Biotechnology Department, Center for Genetic Engineering and Biotechnology, Avenue 31 between 158 and 190, P.O. Box 6162, Havana, 10600, Cuba; d School of Environmental Sciences, University of Guelph, 50 Stone Rd E, Guelph, ON, N1H 2W1, Canada; e Direction of Animal Health, National Center for Animal and Plant Health, Carretera de Tapaste y Autopista Nacional, Apartado Postal 10, San José de las Lajas, 32700, Mayabeque, Cuba; f Institut de Recherche Saint-Louis, Université Paris-Cité, Inserm U976, Team ATIP-Avenir, Paris, France

**Keywords:** Vaccination, Pathogen ecology, Jenner/Pasteur paradigm, Vector-borne pathogen, Pathogen adaptation, Public health strategies

## Abstract

Vaccination campaigns have profoundly influenced the dynamics of infectious diseases, acting as one of the largest ecological experiments in history. By vaccinating billions across decades, we have imposed powerful selective pressures on pathogens, illuminating their ability to adapt, evade, or persist. Rooted in the Jenner/Pasteur paradigm – where exposure to an antigen induces protective immunity – vaccines have revealed how pathogens differ in their ecological susceptibility to immunity. Using this framework, pathogens can be categorized based on their strategies to endure, from those limited by direct immunity to those relying on antigenic variation, chronic infection, or reservoirs. Vector-borne pathogens (VBPs) present a set of challenges to vaccination efforts due to their complex life cycles, stage-specific antigen expression, and reliance on arthropod vectors for transmission. These pathogens not only evade host immunity but also adapt to selective pressures within the vector’s microbiome and immune system. Such complexity often places VBPs beyond the scope of traditional vaccine paradigms, requiring alternative strategies such as transmission-blocking and vector-targeted vaccines. This review explores these insights, examining the interplay between vaccination, pathogen ecology, and evolution – with special emphasis on VBPs – to guide future strategies in vector-borne disease (VBD) control.

## Highlights

• Vaccination campaigns act as large-scale ecological experiments, revealing how pathogens adapt under immune pressure.
• Vector-borne pathogens (VBPs) escaping vaccine-induced immunity expose the limits of the Jenner/Pasteur paradigm.
• Antigenic variation, latency, and environmental persistence enable pathogen survival despite host immunity.
• VBPs adapt to selective pressures within the vector's microbiome and immune system, complicating vaccine development.
• Understanding VBP ecology and evolution highlights the need for vaccine strategies addressing broader ecological dynamics.

## Graphical abstract

**Figure:**
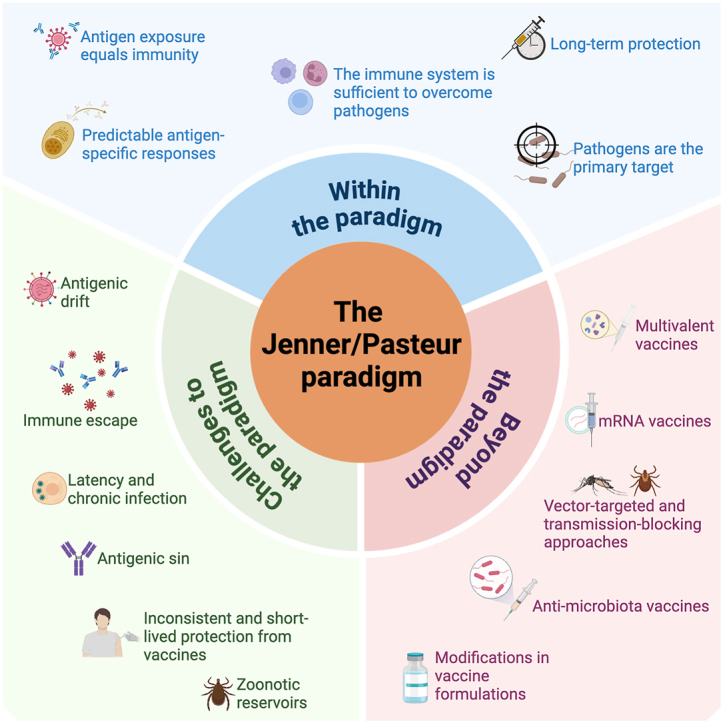

## 1 Introduction

Vaccination represents one of the most profound achievements in biomedical science, transforming global health by dramatically reducing mortality and morbidity from infectious diseases (Pollard and Bijker, 2020). Beyond its immediate benefits, however, vaccination has also unintentionally acted as a massive, real-world experiment in pathogen ecology and evolution. By introducing widespread immunity into human and animal populations, vaccines have imposed powerful selective pressures on pathogens (Plotkin, 2020; Aida et al., 2021; Day et al., 2022). This has revealed intrinsic mechanisms, such as viral proteins that directly interact with the host immune system, and adaption strategies, including escape mutations, that evade immune pressure, shaping their survival, reproduction, and transmission strategies (Meijers et al., 2023; Belman et al., 2024). This ongoing global intervention offers unprecedented insights into how pathogens adapt to their changing environments.

Central to this ecological experiment is the Jenner/Pasteur paradigm, which posits that exposure to antigens through vaccination induces protective immunity, preventing infection or severe disease. While this paradigm has proven transformative, the extent to which pathogens succumb to or circumvent vaccine-induced immunity varies widely (Uraki et al., 2023; Rappuoli et al., 2024). For example, the variola virus causing smallpox lacked the ecological flexibility to persist under vaccine pressure, enabling its eradication (Belongia and Naleway, 2003; Henderson, 2011). In contrast, pathogens such as influenza virus and dengue virus (DENV) have evolved strategies such as antigenic variation or immune escape, though antigenic sin or immune modulation by viral proteins, to maintain their ecological foothold despite widespread vaccination efforts or natural exposures (Henry et al., 2018; St. John and Rathore, 2019).

Furthermore, vector-borne diseases (VBDs) are caused by pathogens transmitted by vectors, usually arthropods such as mosquitoes, ticks, and sand flies. Each year, more than 700,000 deaths are attributed to VBDs, which account for approximately 17% of the global burden of all infectious diseases (WHO, 2024a). In tropical and subtropical regions, these diseases represent a major threat to both human and animal health, an issue further exacerbated by factors such as climate change, urbanization, and population movement (Chala and Hamde, 2021; Hiscox et al., 2025). Malaria, one of the most well-known VBDs, resulted in nearly 263 million cases and 597,000 deaths worldwide in 2023, according to the WHO’s *World Malaria Report 2024*; WHO, 2024b).

The growing incidence of vector-borne pathogens (VBPs) highlights the urgent need for effective strategies to control these diseases. In the past, vaccines have proven to be an effective method that has led to the control of diseases such as smallpox and polio; however, the development of vaccines against VBPs has been far more complex and limited in success (Greenwood, 2014; Centlivre and Combadière, 2015). This difficulty is largely due to the unique and multifaceted biology of VBPs, which involves intricate life cycles, stage-specific antigen expression, and highly evolved immune evasion mechanisms (Palmer et al., 2009; Onyango et al., 2020; Rana et al., 2023). Moreover, effective vaccine design must take into account not only the pathogen itself but also the biology of the vector and its interactions with both the host and the vector’s microbiome, factors that place VBPs outside the scope of traditional vaccine development paradigms. These challenges underscore the necessity for innovative approaches to vaccine design that account for the ecological, molecular, and immunological complexities of VBD transmission.

Moreover, this interplay between immune pressure and pathogen ecology highlights a critical question: What determines a pathogen’s susceptibility or resistance to immunity at the individual and the population level? Vaccination campaigns have illuminated a spectrum of pathogen responses, from those constrained by rapid transmission and host dependency to those leveraging chronic infection, antigenic variation, or environmental reservoirs to persist. These diverse ecological strategies reveal how pathogens adapt to survive under immune pressure.

This review explores how vaccination informs our understanding of VBP ecology and evolution. Here we focus on VBPs transmitted by hematophagous arthropods, primarily mosquitoes and ticks. By evaluating the selective pressures exerted by vaccines and the adaptive responses of VBPs, we aim to uncover key immune evasion strategies, major barriers to effective vaccine development, and innovative approaches for vaccine development that extend beyond the classical Jenner/Pasteur paradigm. A comprehensive understanding of these dynamics is critical for advancing rational vaccine design and strengthening public health strategies against VBDs.

## 2 The Jenner/Pasteur paradigm

**Fig. 1:**
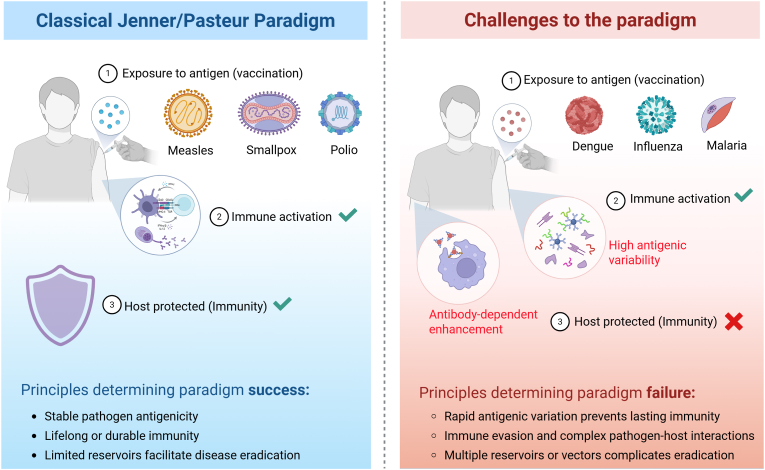
Success and challenges of the Jenner/Pasteur paradigm in vaccination. This figure contrasts the Classical Jenner/Pasteur paradigm with the challenges posed by vaccine-resistant pathogens. On the left, the Jenner/Pasteur paradigm shows how vaccines against stable pathogens like measles, smallpox, and polio lead to immune activation, generating lasting immunity and effective disease control. Success relies on stable antigenicity, durable immunity, and limited reservoirs. On the right, challenges arise with pathogens like dengue, influenza, and malaria, which exhibit high antigenic variability and immune evasion. Despite immune activation, these pathogens often undermine immunity through rapid mutation and antibody-dependent enhancement (ADE), resulting in incomplete protection. Success is limited by rapid antigenic variation, complex pathogen-host interactions, and multiple reservoirs or vectors, making eradication difficult.

The Jenner/Pasteur paradigm has long served as the foundation of vaccinology, built on the principle that exposure to a pathogen – or its antigens through vaccination – trains the immune system to recognize and combat future infections. This concept, first demonstrated through Edward Jenner’s use of cowpox to protect against smallpox and later expanded by Louis Pasteur with the development of vaccines against rabies and anthrax, revolutionized infectious disease prevention (Plotkin, 2014). The assumption that controlled antigen exposure can generate durable and protective immunity underpins nearly all modern vaccines (Fig. 1) (Rappuoli et al., 2011).

A fundamental tenet of this paradigm is that vaccination induces predictable, antigen-specific immune responses capable of conferring long-term protection. By generating antibodies and memory T and B cells, vaccines provide immunity that can persist for years or even a lifetime, as seen with smallpox and measles vaccines (Pollard and Bijker, 2020). This immunological memory ensures a rapid and robust response upon subsequent exposure to the pathogen. Another assumption embedded in this model is that the immune system, once primed by vaccination, is sufficient to clear or control infections. Consequently, vaccine development has historically focused on leveraging natural immune mechanisms to elicit protective immunity, with the expectation that an effective immune response would be enough to halt pathogen transmission and prevent disease progression (Rappuoli et al., 2024).

While this approach has successfully controlled many infectious diseases, it does not universally apply. The Jenner/Pasteur paradigm assumes that pathogens are the primary targets of immunity and that their elimination through vaccination will prevent disease. However, many pathogens have evolved sophisticated mechanisms to evade immune detection, persist in the host, or rapidly adapt under vaccine-induced selective pressures (Fig. 1). This is evident in the case of rapidly mutating viruses like influenza, which necessitate frequent vaccine updates due to antigenic drift and shift (Petrova and Russell, 2017; Han et al., 2023), or HIV, which effectively suppresses immune responses to maintain persistent infection (Yang et al., 2003). Similarly, *Mycobacterium tuberculosis* and *Plasmodium falciparum* have developed strategies to evade immune clearance, contributing to the inconsistent efficacy of vaccines against tuberculosis and malaria (Martinez et al., 2022; Duffy et al., 2024).

Another challenge to the paradigm arises from pathogens that do not rely solely on direct host-to-host transmission. VBDs such as malaria and dengue, which depend on mosquito vectors, cannot be eradicated through vaccination alone. These pathogens exist within complex ecological networks where targeting the pathogen itself is insufficient without also addressing its transmission dynamics (Wilson et al., 2020). Additionally, some pathogens persist in environmental or animal reservoirs, complicating efforts to eliminate them through traditional vaccination strategies (Rupprecht et al., 2022).

Moreover, the immune responses elicited by vaccines do not always follow the predictable patterns assumed by the Jenner/Pasteur model. The phenomenon of antibody-dependent enhancement (ADE) in DENV infection illustrates how pre-existing immunity can paradoxically worsen disease upon subsequent exposure to related viral strains (Guzman and Vazquez, 2010). Likewise, original antigenic sin, where immune responses are skewed toward previously encountered antigens at the expense of generating new, variant-specific responses, has been observed in both influenza and SARS-CoV-2 infections, complicating vaccine efficacy (Vatti et al., 2017; Aguilar-Bretones et al., 2023).

Despite these challenges, the Jenner/Pasteur paradigm remains a cornerstone of immunology, providing the basis for many of the world’s most successful vaccines. However, the increasing recognition of pathogen adaptation and immune evasion necessitates an expanded perspective that integrates ecological and evolutionary principles into vaccine design. Understanding how pathogens, particularly VBPs, persist under immune pressure and refining strategies to counteract their evasion mechanisms will be critical in overcoming the limitations of traditional vaccine approaches. The next sections will explore these challenges in greater detail, emphasising the methods by which VBPs evade vaccine-induced immunity, the obstacles currently hindering vaccine development, and the alternative strategies that may be employed in the future to control VBDs.

## 3 Vector-borne pathogens evading vaccine-induced immunity under the Jenner/Pasteur paradigm

The success of the Jenner/Pasteur paradigm, with its core tenets of antigen exposure and immune memory, has revolutionized public health by reducing or eliminating the burden of many infectious diseases (Rappuoli et al., 2011; Plotkin, 2014). Yet, the paradigm has its limitations. Certain pathogens, particularly VBPs, resist or circumvent vaccine-induced immunity (Gandon et al., 2001), challenging assumptions about how exposure leads to protection. These pathogens exploit unique biological and ecological strategies, revealing gaps in our understanding of protective immunity and the interplay between host and pathogen (Bhattacharjee et al., 2023; Rana et al., 2023). Examining these challenges sheds light on the limitations of the paradigm and the need for innovative approaches to vaccine development.

**Fig. 2:**
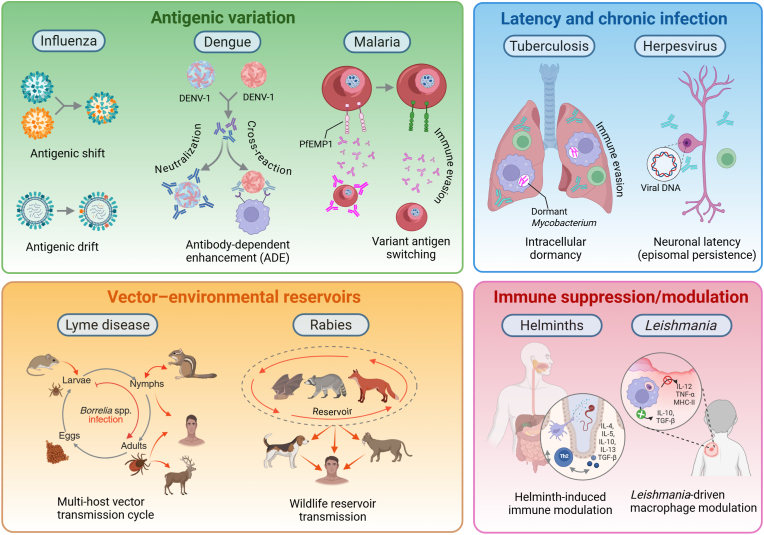
Pathogen strategies to evade vaccine-induced immunity. Pathogens use diverse mechanisms to persist despite immune pressure. Antigenic variation (*green*) allows pathogens like influenza (*via* antigenic drift and shift), dengue (through antibody-dependent enhancement), and malaria (by switching surface antigens) to evade immune detection. Latency and chronic infection (*blue*) enable pathogens such as *Mycobacterium tuberculosis* (intracellular dormancy) and herpesvirus (neuronal latency) to persist undetected. Vector-environmental reservoirs (*orange*) sustain diseases like Lyme disease (through multi-host tick cycles) and rabies (*via* wildlife reservoirs). Immune suppression/modulation (*red*) helps helminths (by inducing regulatory cytokines) and *Leishmania* (by altering macrophage responses) to avoid immune clearance. These strategies reflect the complex ecological and evolutionary adaptations pathogens use to evade immunity and challenge vaccine effectiveness.

One of the foundational assumptions of the Jenner/Pasteur paradigm is that exposure to antigens – whether through infection or vaccination – induces immune memory that confers long-term protection (Cabezas-Cruz et al., 2025). This principle underpins most modern vaccines (Rappuoli et al., 2024), yet some pathogens resist this mechanism entirely. For instance, viruses such as HIV and hepatitis C virus (HCV) evade immune memory by mutating rapidly, rendering early antibody and T cell responses rapidly ineffective (John and Gaudieri, 2014). In the case of HIV, antigenic variation and the ability of the virus to suppress the immune system make it exceedingly difficult for vaccines to induce durable, protective responses (Fig. 2) (Yang et al., 2003). Similarly, influenza’s antigenic drift and occasional antigenic shift necessitate annual updates to vaccines (Fig. 2) (Han et al., 2023), as the virus rapidly evolves to escape immune detection. Likewise, pathogens such as *Neisseria gonorrhoeae* avoid immune recognition altogether by altering their surface antigens, allowing for repeated infections despite prior immune responses (Walker et al., 2023). For these pathogens, traditional vaccine approaches based on stable antigen presentation fail to deliver the expected long-term immunity.

Compounding this complexity is the concept of antigenic sin (Vatti et al., 2017) (also known as original antigenic sin), where the immune system preferentially recalls memory responses to previously encountered antigens rather than generating novel responses to new but related antigens. For example, with influenza, prior exposure to one strain can skew immune responses toward epitopes shared with earlier strains, reducing the efficacy of responses to the new variant (Henry et al., 2018; Aguilar-Bretones et al., 2023). Similarly, SARS-CoV-2 variants have demonstrated that pre-existing immunity from earlier strains or vaccines may sometimes provide suboptimal protection or even hinder immune adaptation to new variants (Aguilar-Bretones et al., 2023). These examples demonstrate that immune responses are not always beneficial or predictable, particularly for pathogens with evolving antigenic profiles.

VBPs add additional layers of complexity, shaped by transmission ecology and evolutionary pressure from both hosts and vectors. Many VBPs exploit immune evasion strategies analogous to HIV or influenza, but with added challenges of antigenic diversity across strains, life-cycle stages, or geographical regions. For example, *Trypanosoma vivax* and *Trypanosoma brucei*, transmitted by biting flies and tsetse flies, respectively, rely on dense coats of variant surface glycoproteins (VSGs) that switch periodically to stay ahead of host antibody responses (Jackson et al., 2012). Their vast VSG gene repertoires enable immune evasion over prolonged infections. *Anaplasma marginale*, a tick-borne rickettsial bacterium of cattle, undergoes continuous antigenic variation of its Msp2 surface protein, driven by gene conversion events, thwarting both natural and vaccine-induced immunity (Palmer et al., 2009, 2016). *Babesia* species, tick-borne pathogens closely related to *Plasmodium* spp., display stage-specific antigenic variation and immune suppression mechanisms, contributing to chronicity and relapse (Allred, 2001; Djokic et al., 2021; Puri et al., 2021). Moreover, *Borrelia burgdorferi*, the causative agent of Lyme disease, undergoes antigenic variation of its VlsE surface lipoprotein within the mammalian host, enabling persistent infection despite adaptive responses (Zhang et al., 1997; Anderson and Brissette, 2021). Analyses of the *Theileria annulata* surface protein (TaSP) and the polymorphic immunodominant molecule (PIM) of *Theileria parva* have identified genetic recombination as a key mechanism driving antigenic diversity in these pathogens, possibly enabling them to evade host immune responses (Schnittger et al., 2002; Geysen et al., 2004; Sivakumar et al., 2014)*.* Additionally, studies have demonstrated that vaccination against *T. parva* induces strain-specific immunity, predominantly mediated by major histocompatibility complex (MHC) class I-restricted CD8^+^ T cells. These findings highlight how the evolutionary dynamics of genetic diversification facilitate immune evasion in *T. parva*, posing significant challenges to the development of broadly protective vaccines (Connelley et al., 2011; Sivakumar et al., 2014; Amzati et al., 2019). In each case, these pathogens co-opt variation and immune escape to persist over time, in both hosts and vectors, rendering one-time vaccination insufficient.

Among VBDs, malaria caused by *P. falciparum* presents one of the most significant challenges to vaccine development. The RTS,S vaccine targets the circumsporozoite protein (CSP) of *P. falciparum*, specifically the central repeat and C-terminal regions (Zavala, 2022). It consists of a fusion protein containing part of the CSP and the hepatitis B surface antigen, and is formulated with the AS01 adjuvant to enhance immunogenicity (RTSS Clinical Trials Partnership, 2015). While RTS,S induces antibody and CD4^+^ T cell responses, its efficacy is modest, around 30–50% in children, and declines within months after vaccination (RTSS Clinical Trials Partnership, 2015; White et al., 2015). The limited efficacy is due in part to *P. falciparum* antigenic polymorphism, immune evasion mechanisms like CSP variation, and the parasite’s complex life cycle that includes both pre-erythrocytic and blood stages (Khan et al., 2019; Dieng et al., 2023; Mitchell et al., 2024). The inability of RTS,S to generate durable, sterilizing immunity highlights the difficulty of targeting parasites that can rapidly adapt or hide from immune surveillance. Additionally, vector behavior and transmission intensity complicate deployment, necessitating integration with vector control strategies for meaningful impact. Next-generation malaria vaccines, such as R21/Matrix-M (Datoo et al., 2024) and transmission-blocking vaccines (TBVs) (e.g. targeting Pfs25, Pfs230 or Pfs48/45), aim to overcome these limitations through multivalent designs, targeting multiple life stages, or focusing on conserved sexual-stage antigens (Draper et al., 2018). Nevertheless, their success depends on addressing the immune escape dynamics that are characteristic of VBPs.

The assumption that immunological memory equates to long-term protection is also challenged by pathogens that exploit gaps in immune longevity. For example, the *Mycobacterium bovis* BCG vaccine, while protecting against severe tuberculosis in children, offers inconsistent and short-lived protection against pulmonary tuberculosis in adults (Martinez et al., 2022). Likewise, RTS,S protection is transient, reinforcing the notion that memory alone cannot outmatch immune evasion in pathogens that exploit latency or complex antigenic structures (Mitchell et al., 2024). These examples highlight the inadequacy of relying solely on immunological memory to control pathogens that either persist in latent forms or exploit the host’s inability to maintain durable immune responses.

Perhaps the most profound challenge to the paradigm lies in its assumption that the immune system, once trained by vaccination, is sufficient to overcome pathogens. This assumption is particularly problematic for many VBPs, which have evolved to persist in the host by evading or suppressing adaptive immunity. For example, *Leishmania* spp., transmitted by sand flies, evade immune clearance by residing within macrophages and manipulating host cytokine responses to promote regulatory and anti-inflammatory environments (Heyde et al., 2018; da Silva et al., 2021). *Plasmodium falciparum* also exhibits strategies for immune evasion and persistence. The parasite’s ability to sequester in tissues, vary surface proteins like PfEMP1, and alter expression of immunomodulatory molecules helps it persist during blood-stage infections and contributes to clinical relapses or incomplete immunity despite repeated exposure (Sakoguchi and Arase, 2022).

An often overlooked yet critical aspect of vaccine design for VBPs is tissue-specific immunity at the site of pathogen entry. While most mucosal vaccines are developed for pathogens that enter through the respiratory or gastrointestinal tracts, many VBPs are introduced through the skin by the bite of an infected arthropod. Although the skin is not a mucosal surface, it harbors a rich immune network – including Langerhans cells, dermal dendritic cells, and tissue-resident memory T cells – that can mediate rapid, localized immune responses (Kabashima et al., 2019). Some vectors actively modulate the host immune response during transmission. For instance, mosquito saliva contains proteins that bind immune receptors, recruit susceptible myeloid cells, induce autophagy, and suppress cytokine production, collectively enhancing arbovirus establishment in the skin (Wang et al., 2024b). Targeting this compartment through intradermal delivery, microneedle patches, or live-attenuated vectors introduced *via* the skin offers a promising strategy for enhancing vaccine efficacy against pathogens like *Leishmania* spp., *Trypanosoma* spp., *Plasmodium* spp., and *Borrelia* spp., all of which initiate infection at the dermal level (Voza et al., 2010; Louis et al., 2019; Korkmaz et al., 2020).

In parallel, mucosal immunity may still play a role in VBDs that spread to or through mucosal tissues after initial skin entry. For instance, Zika virus (ZIKV) and some arboviruses can be transmitted sexually and persist in mucosal sites, while *Plasmodium* spp. and *Trypanosoma* spp. eventually reach organ systems where mucosal surfaces contribute to immune regulation (Giddings et al., 2006; Counotte et al., 2018). Enhancing local IgA production, recruiting tissue-resident T cells, and priming mucosa-associated lymphoid tissues (MALT) could support systemic immunity and help contain secondary dissemination. These approaches parallel advances seen in respiratory pathogens like SARS-CoV-2 and influenza, where intranasal vaccines not only reduce viral transmission but also enhance cross-reactive immunity, potentially providing broader protection against antigenically drifted strains (Singh et al., 2023; Ying et al., 2024). In contrast, systemic vaccines administered *via* intramuscular or subcutaneous routes often elicit weak or insufficient mucosal immune responses (Holmgren and Czerkinsky, 2005). A better understanding of both cutaneous and mucosal immune niches in the context of vector transmission may enable the design of vaccines that intercept pathogens before they establish persistent infection.

Finally, the paradigm often focuses narrowly on the pathogen itself, overlooking the broader ecological and environmental contexts in which transmission occurs. This limitation is especially evident in vector-borne and zoonotic diseases. For example, dengue (King et al., 2020) and malaria (Msugupakulya et al., 2023) depend on mosquito vectors, while rabies (Rupprecht et al., 2022) persists in animal reservoirs (Fig. 2). Vaccines targeting these pathogens alone cannot eliminate disease (Wilson et al., 2020); instead, they must be integrated with vector control strategies or animal vaccination campaigns to reduce transmission effectively. This broader perspective highlights the need to move beyond the host-pathogen relationship to include ecological dynamics in vaccine strategies.

Pathogens that resist vaccine-induced immunity, especially VBPs, compel us to move beyond the classical assumptions of antigen exposure and memory. Their success hinges on sophisticated immune evasion strategies, from antigenic variation and latency to tissue-specific suppression and ecological persistence. While the Jenner/Pasteur paradigm remains foundational, it must be expanded into a more holistic framework that incorporates tissue-resident immunity, antigenic diversity, ecological dynamics, and systems vaccinology. Addressing these challenges will require a shift toward flexible, multivalent, and context-aware vaccines, tailored not just to pathogens, but to the dynamic landscapes in which they evolve and spread.

## 4 Ecological insights from vector-borne pathogens escaping vaccines guided by the Jenner/Pasteur paradigm

The successes and limitations of vaccines guided by the Jenner/Pasteur paradigm reveal profound insights into pathogen ecology. The evasion of vaccine-induced immunity by pathogens is not a random occurrence but rather the result of evolved strategies that ensure their persistence in immunized hosts (Gandon et al., 2001). These adaptive mechanisms are integral to a broader set of survival strategies shaped by evolutionary pressures arising from pathogen interactions with both the host and the environment. Among these strategies, pathogens escape vaccine-induced immunity through latency, chronic infection, antigenic variation, and immune adaptation within the host, allowing them to persist and evade clearance despite immunological pressures (Mustafa, 2024).

For VBPs, vaccine evasion presents an additional challenge, as these microorganisms must also contend with selective pressures imposed by direct interaction with the vector’s microbiome and immune system (Shaw and Catteruccia, 2018; Lewis et al., 2023; Ratcliffe et al., 2024). Within arthropods, pathogens develop diverse ecological strategies that enhance their ability to adapt, survive, and evolve within the vector, ultimately favoring their persistence and efficient transmission. Examining the ecology and evolution of pathogens, particularly their ability to evade the Jenner/Pasteur paradigm by adapting to host-environment interactions and vector-driven evolutionary pressures, provides valuable insights into infections persistence. This understanding helps develop more effective strategies to control infectious diseases, especially those transmitted by vectors.

Vector-borne pathogens face additional selective pressures imposed by the vector’s biological and ecological environment. These microorganisms could evolve toward more virulent genotypes and phenotypes as a result of interactions with the vector to facilitate colonization and subsequent transmission (De Angeli Dutra et al., 2022). These adaptations include altered community dynamics through interactions with resident microbiota, evasion of the vector’s immune defenses, and metabolic adjustments to survive under fluctuating physiological conditions.

A broader ecological perspective reveals that antigenic variation is a fundamental strategy used by *P. falciparum* (Barcons-Simon et al., 2023), and *B. burgdorferi* (Chaconas et al., 2020) to bypass immune memory. In densely connected host populations where frequent re-exposure occurs, rapid genetic evolution ensures survival by generating antigenic diversity. These cases demonstrate how strong selective pressures from the immune system, whether induced by natural infection or vaccination, drive the continuous evolution of pathogens. By constantly reshuffling their genetic composition, these organisms maintain their foothold despite widespread immunity. This strategy is particularly effective in pathogens with high mutation rates or genetic recombination capabilities, such as *P. falciparum*. Ultimately, these pathogens illustrate that evolutionary flexibility can be just as crucial as ecological stability in ensuring long-term persistence.

On the other hand, rather than relying on accelerated evolution to generate antigenic diversity, tick-borne pathogens such as *A. phagocytophilum* adopt ecological adaptations to optimize their colonization within host populations and vectors (Stuen et al., 2013; Aardema and Von Loewenich, 2015). These adaptations involve the selection of variants best suited to specific environmental conditions and transmission dynamics. Using phylogenetic and network analyses, Jaarsma et al. (2019) identified 199 haplotypes distributed across four ecotypes, each with specific interactions between ticks, vertebrates, and geographical regions. The results suggested that selective pressures from the vector and the environmental setting influence the evolution of the pathogen, with significant variations in rodent communities in specific regions compared to others. The reduction in microbial diversity and the displacement of taxa favor its establishment within the vector, ensuring its transmission. These findings highlight that pathogens not only evolve to evade the host immune response but also to adapt to the ecological conditions of the vector, which can impact their persistence and speciation.

Furthermore, pathogens within arthropod vectors interact with the resident microbial community in ways that can be either cooperative or competitive, thereby directly shaping their colonization and transmission dynamics (Gupta and Nair, 2020; Pavanelo et al., 2023). These interactions follow the hypotheses that pathogens actively alter microbiota composition, reducing biodiversity and disrupting microbial cooperation. As in other ecosystems, by inhibiting biofilm formation and diminishing mutualistic interactions such as cross-feeding and syntropy, they could compromise the functional stability of the microbial community, creating conditions that favor their persistence (Nadell et al., 2016; Weiland-Bräuer, 2021; Sarsan et al., 2022).

The persistence of a pathogen in a host often shapes its adaptive strategies within the vector, optimizing conditions for transmission. Once inside the vector, the pathogen interacts with the resident microbiota, influencing microbial diversity and modulating exclusion or facilitation mechanisms that impact its establishment. Over time, selective pressures refine its position within the community, favoring the emergence of variants with enhanced transmissibility (Dennison et al., 2014; Wang et al., 2023). For example, *A. marginale* has developed mechanisms that optimize its colonization within *Rhipicephalus microplus*, thereby ensuring its transmission and persistence. Its prolonged presence in cattle without clinical signs post-infection could lead to a potential adaptation within the vector, facilitating its replication (Piloto-Sardiñas et al., 2023). As the infection progresses, *A. marginale* reduces microbial diversity, displacing taxa through ecological competition, thereby progressively establishing itself within the *R. microplus* microbiome (Piloto-Sardiñas et al., 2024). Furthermore, interaction with the vector’s microbiota can influence its acquisition, affecting transmission through factors such as cyclical antigenic variation, vector immunity, and microbial competition at the feeding site. Over time, *A. marginale* forms independent clusters, reinforcing its position and favoring variants with greater transmissibility (Piloto-Sardiñas et al., 2024). These findings demonstrate that its adaptation to the vector depends not only on immune evasion but also on active modification of the microbiome to consolidate its presence and maximize its transmission success (Piloto-Sardiñas et al., 2023, 2024). These adaptive mechanisms reshape the microbial landscape, ultimately creating an environment that favors the establishment and long-term survival of *A. marginale* within the vector.

As part of their ecological adaptation during colonization, pathogens in hard ticks can induce metabolic shifts within the microbial community, disrupting functional cooperative dependencies and altering microbial interactions (Abraham et al., 2017; Adegoke et al., 2020; Maitre et al., 2022). Specifically, *A. phagocytophilum* modifies the physiology of the vector and, consequently, the microbiota to facilitate its colonization in *Ixodes scapularis* (Abraham et al., 2017). This mechanism induces the expression of an antifreeze glycoprotein that inhibits biofilm formation, altering the functional relationships within the microbial community and favoring its persistence. In addition, the presence of *A. phagocytophilum* in the vector causes fragmentation of metabolic pathways, promoting greater compactness and functional specialization in associated microorganisms, which reinforces their survival (Paulino et al., 2024). By reducing functional dependencies and altering metabolic interactions, *A. phagocytophilum* manipulates the vector’s gut microbiome, ensuring favorable conditions for its transmission (Abraham et al., 2017; Paulino et al., 2024).

Like bacterial pathogens, viruses undergo evolutionary mechanisms as a result of interaction with their vectors that favor their replication and subsequent transmission (Öhlund et al., 2019). Despite structural and pathogenic differences, viral load in many environments is largely determined by the abundance and metabolic activity of the bacterial community (Lima et al., 2019). In mosquitoes, changes in bacterial composition have been shown to enhance or inhibit viral transmission. Consequently, viruses have developed strategies to modulate the behavior of the resident bacterial community to enhance their colonization. West Nile virus (WNV) has evolved mechanisms that optimize its transmission within *Culex* mosquitoes, adapting to the vector’s microbiota and modulating its immune system. Its ability to actively suppress *Wolbachia* populations, either by direct inhibition or by regulating the Toll and IMD immune pathways, demonstrates a key evolutionary strategy to prevent this bacterium from interfering with its replication (Garrigós et al., 2023). Furthermore, WNV alters the bacterial composition of the vector, promoting an increase in *Enterobacter* and *Serratia*, which favors its persistence by modifying the mosquito’s intestinal environment. It also regulates the expression of microRNAs (miRNAs), interfering with the vector’s antiviral responses to facilitate its replication and transmission (Hussain et al., 2011). These adaptive mechanisms demonstrate that WNV not only evades host immunity but also evolves within the vector to manipulate its microbiome and immune response, ensuring its survival and spread.

Additionally, arboviruses have evolved mechanisms that allow them to persist in vectors and optimize their transmission by manipulating mosquito immunological and metabolic pathways. One of the main mechanisms is the modulation of the RNAi pathway, where viruses such as ZIKV regulate the expression of small interfering RNAs (siRNAs), Piwi-interacting RNAs (piRNAs), and miRNAs to interfere with the vector’s antiviral response and promote its replication. Silencing key enzymes such as Argonaute-2 and Dicer2/R2D2 increases the replication of flaviviruses (i.e. DENV) and alphaviruses (i.e. chikungunya virus [CHIKV]) in *Aedes* spp. and *Anopheles* spp., demonstrating an adaptation that allows for greater transmission efficiency (Blair and Olson, 2015; Liu et al., 2019).

Another important evolutionary mechanism is the modification of vector metabolism, as demonstrated by DENV-2 and CHIKV, which induce antioxidant responses in the mosquito midgut. This leads to activation of detoxification proteins that facilitate viral persistence (Tchankouo-Nguetcheu et al., 2010; Santana-Román et al., 2025). Additionally, both viruses manipulate carbohydrate and lipid metabolic pathways to create a physiologically favorable environment for replication, thereby enhancing transmission efficiency. These infections are associated with increased expression of proteins involved in reactive oxygen species (ROS) generation, energy production, and metabolic regulation. Collectively, such viral strategies aim to maintain cell viability and promote sustained infection within the vector (Santana-Román et al., 2025).

Other strategies developed by arboviruses to alter vector immunity include the suppression of the JAK-STAT and IMD pathways, which increase viral replication in certain *Aedes aegypti* strains (McFarlane et al., 2014; Sim et al., 2014). Additionally, processes such as autophagy and apoptosis may contribute to antiviral defense, as they compromise cell viability. Therefore, arboviruses, both in hosts and vectors, direct their evolutionary strategies toward inhibiting these processes in order to maintain cell integrity and thus complete their replication cycle (Mehrbod et al., 2019; Cappuccio and Maisse, 2020; Chowdhury et al., 2020).

The mechanisms of evolution within the vector allow pathogens to continue circulating in ecosystems, even when the host population has been immunized with traditional vaccines developed under the Jenner/Pasteur paradigm. Therefore, to achieve effective eradication, it is necessary to complement immunization with strategies that interfere with the evolution and ecology of the pathogen within the vector.

## 5 Challenges in vaccine development for vector-borne pathogens

The Jenner/Pasteur paradigm has traditionally focused on the pathogen itself, without taking into account the broader ecological and environmental contexts in which transmission occurs, for instance, in vector-borne and zoonotic diseases. VBPs often rely on vector’s mechanisms to evade host immune response to facilitate transmission. Additionally, vectors can act as reservoir of these pathogens, promoting their persistence and contributing to disease re-emergence, which complicates control efforts (Mackenzie and Jeggo, 2013; Berube, 2023). Considering the limitations posed by VBP control that escape the paradigm, alternative vaccine strategies have been proposed, such as transmission-blocking and vector-targeted vaccines. These approaches focus on disrupting pathogen transmission rather than solely eliciting host immunity against the pathogen, moving beyond the classical pathogen-centered model (Neelakanta and Sultana, 2015).

A major challenge in developing vaccines against these pathogens lies in their complex life cycles, our limited understanding of protective immune mechanisms, and their highly evolved immune evasion strategies. VBPs have developed various mechanisms to evade and manipulate host immune responses, allowing them to persist and, in some cases, establish chronic infections. These immune evasion strategies include avoiding host recognition, modulating of surface components (antigenic variation), inhibiting of the host complement pathway, neutralizing antimicrobial molecules, suppressing antiviral gene induction, and interfering with host cell defence pathways and cytokine production (Bhattacharjee et al., 2023; Rana et al., 2023; Alghamdi et al., 2025). Such mechanisms present a substantial barrier to vaccine development, and are described in detail in the previous sections of this review.

VBPs exhibit multi-stage life cycles involving both vertebrate hosts and invertebrate vectors, as seen in pathogens such as species of *Leishmania*, *Plasmodium*, and *Trypanosoma* (Wheeler et al., 2011; Kuleš et al., 2016; Dvorin and Goldberg, 2022; Martín-Escolano et al., 2022). Each stage expresses a unique set of antigens, making it difficult to identify vaccine targets that can induce broad and cross-stage protection (Stanberry and Strugnell, 2011; Alzan et al., 2024). For example, *P. falciparum* undergoes approximately ten morphological transitions across five distinct host tissues during its two-host life cycle, with each stage expressing different major proteins, rendering antigen targets highly stage- and host-specific (Mackinnon and Marsh, 2010; Stanberry and Strugnell, 2011). Similarly, species of *Babesia* and *Theileria* develop sexual stages, kinetes and sporozoites within tick vectors, then invade the erythrocytes of vertebrate hosts. Hence, targeting multiple parasite developmental stages, such as blood and sexual stages, may be necessary for effective vaccination strategies (Alzan et al., 2024).

Another important difficulty in the development of vaccines against VBPs is the lack of suitable animal models that can accurately reproduce the full complexity of pathogen life cycles, including aspects of pathogenesis, pathogen-host-vector interactions, immunological responses, vaccine efficacy, and *in vivo* maintenance of the pathogen (Teixeira and Gomes, 2013; Gerdts et al., 2015). Various laboratory models do not fully mimic the physiological and immunological characteristics of natural hosts or replicate the critical vector-host-pathogen interface, which limits the extrapolation of experimental results to clinical settings (Tesfamariam et al., 2022; Olajiga et al., 2024). For instance, current animal models for *Orientia tsutsugamushi*, which cause the scrub typhus disease, do not reproduce key features of the human disease, such as systemic endothelial damage and the percutaneous transmission route *via* trombiculid mite bites, features essential for understanding disease pathogenesis. These gaps hamper efforts progress in vaccine development against this pathogen (Díaz et al., 2018; Kim, 2018; Sarathy and Walker, 2020). Additionally, maintaining certain bacterial pathogens, such as *Rickettsia* and *Anaplasma* species, in laboratory settings is technically challenging because these bacteria are obligate intracellular organisms that require specialized eukaryotic cell cultures for replication (Salata et al., 2021).

Moreover, results obtained from *in vitro* systems may not accurately represent *in vivo* pathogen behaviour, host immune modulation, or vector-specific interactions, all of which are essential for rational vaccine design (Manning et al., 2018; Salata et al., 2021). In preclinical studies, direct needle injection of a virus into an experimental host bypasses the important biological role of the vector in facilitating pathogen transmission. This may partly explain why certain candidate vaccines show efficacy in controlled laboratory environments but fail when tested under field-like or natural transmission scenarios (Peters et al., 2009; Thangamani et al., 2010; Manning et al., 2018). Therefore, animal models that replicate natural transmission routes, such as mosquito bites or the co-inoculation of saliva with the pathogen, are preferable, particularly when evaluating the immunogenicity of vaccine candidates in advanced stages of preclinical development (Manning et al., 2018). However, working with arthropod vectors in laboratory facilities introduces additional logistical and biosafety complexities, which constrain the capacity to study certain pathogen developmental stages or transmission dynamics under controlled experimental conditions (Tabachnick, 2006; Qian, 2017).

Vector saliva, delivered during blood-feeding, has been shown to exacerbate infections caused by various vector-borne viruses, including DENV and WNV, by modulating viral replication and pathogenesis (Styer et al., 2011; Hastings et al., 2019). In *Leishmania* infections, the sandfly saliva contains components that interfere with blood coagulation or modulate host immunity, either promoting immune evasion by the pathogen or triggering specific host immune responses (Volpedo et al., 2021). These findings have encouraged the development of vector-targeted vaccines, which aim to disrupt vector physiology or block molecular pathways essential for pathogen transmission, thereby reducing infection risk in the vertebrate host (Kuleš et al., 2016; de la Fuente et al., 2017; Alzan et al., 2024). Nevertheless, developing effective vaccines targeting arthropod vectors remains highly challenging, these multicellular eukaryotic organisms possess complex and multi-stage life cycles, making it difficult the identification of conserved, protective antigens. In addition, vectors express highly variable molecules depending on their species, developmental stage, sex, or environmental conditions, complicating the selection of universal vaccine targets (Oleaga et al., 2021; Abbas et al., 2023; Busch et al., 2025; Guo et al., 2025). In most cases, the only contact between the vector and the host immune system occurs briefly during blood-feeding, limiting the opportunity for the host to mount an effective immune response against vector-derived antigens (Manning and Cantaert, 2019; Thomas et al., 2022).

The remarkable biodiversity of vector species, many of which are capable of parasitizing a wide range of vertebrate hosts, further complicates the development of an effective vaccine (Kumar et al., 2012; Parizi et al., 2023). For example, nearly 900 tick species have been described worldwide, each potentially transmitting different pathogens and exhibiting species-specific biological and molecular traits. This diversity represents a considerable barrier to the development of cross-protective vaccines aimed at controlling tick-borne diseases through immunization. Moreover, variations in feeding behaviour, salivary protein composition, and vector-host specificity make it even more difficult to identify conserved targets suitable for broad-spectrum vector control vaccines (Thomas et al., 2022; da Silva Vaz Junior et al., 2024; Moming et al., 2024; Wang et al., 2024a).

Finally, vaccines against VBDs also face substantial challenges in terms of accessibility and distribution, particularly in countries where these diseases are often endemic. In many of these regions, there is limited governmental investment in vaccine research, development, and production infrastructure. Furthermore, public health policies may lack the necessary prioritization of VBD control due to competing healthcare demands, limited resources, and political or logistical constraints. Even when vaccines are available, challenges such as high production costs, cold chain requirements, inadequate healthcare infrastructure, and difficulties in reaching rural or marginalized populations can hinder widespread immunization coverage. These factors collectively contribute to the persistent burden of VBDs in vulnerable populations and underscore the urgent need for global collaborative efforts to improve vaccine accessibility, affordability, and delivery systems tailored to the realities of affected regions (Orenstein et al., 2014; Okwor and Uzonna, 2016; Bangura et al., 2020).

The growing threat of vector-borne and zoonotic diseases has prompted a broader approach to vaccine development. Nevertheless, significant challenges still persist in creating an accessible, effective and universal vaccine against these pathogens. This highlights the urgent need to deepen our understanding of the molecular interactions at the vector-host-pathogen interface, the role of the microbiome, vector biology, and host immune responses. Moreover, innovative vaccine strategies are needed, ones that move beyond the traditional Jenner/Pasteur paradigm and are tailored to meet contemporary public health demands.

## 6 Vaccine development and alternative strategies beyond the Jenner/Pasteur paradigm

Emerging vaccine strategies aim to address the challenges posed by pathogens resistant to traditional approaches. However, these innovations vary in their adherence to or departure from the Jenner/Pasteur paradigm, highlighting both its enduring strengths and its limitations.

Targeting conserved antigens remains within the Jenner/Pasteur paradigm, focusing on stable antigens to elicit immune memory. This approach adheres to the principle that exposure to key antigens induces protective immunity. Examples include universal influenza vaccines targeting the conserved hemagglutinin stalk or non-structural antigens and malaria vaccines targeting invariant parasite proteins (Duffy et al., 2024; Mosmann et al., 2024). The success of these vaccines depends on whether conserved regions can elicit strong and durable immune responses, a challenge for highly immune-evasive pathogens like influenza or HIV.

Another strategy within the paradigm is the development of multivalent and pan-serotype vaccines, which expand the diversity of antigens presented to broaden immune coverage, remaining rooted in the assumption that exposure leads to protective immunity. Dengue vaccines targeting all four serotypes and pneumococcal vaccines covering dozens of capsular types are some examples based on this strategy (Kallas et al., 2020; Musher et al., 2022). However, the effectiveness of the multivalent vaccines may be undermined by antigenic variation outside the targeted set, as seen with evolving serotypes in pneumococcus (Feldman and Anderson, 2014).

Furthermore, modifications in the vaccine formulations extend beyond the Jenner/Pasteur paradigm. Some approaches include the use of antigenic vehicles and/or the incorporation of immune stimulants (e.g. adjuvants), as well as the administration routes to amplify, prolong or elicit immune responses in specific body sites, recognizing that antigen exposure alone may be insufficient. For example, adjuvanted tuberculosis or RTS,S/AS01 malaria vaccines to enhance T-cell responses and HIV vaccines aimed to produce broadly neutralizing antibodies (Enriquez et al., 2021; Haynes et al., 2022; Nadeem et al., 2022). Additionally, nanoparticle-based strategies have also been explored. For instance, a multivalent vaccine against Lyme disease was developed using the outer surface protein A (OspA) from *Borrelia*, fused to bacterial ferritin to form self-assembling nanoparticles. This vaccine showed durable antibody response in mice and non-human primates (Kamp et al., 2020). Nevertheless, this strategy still relies on eliciting immune memory, leaving unresolved challenges for pathogens that evade or suppress immunity.

For pathogens that escape the paradigm, achieving protective immunity is no longer the primary goal of vaccination; instead, the focus has shifted to preventing severe disease while allowing controlled circulation of the pathogen within the population. This shift is particularly relevant for highly transmissible viruses, such as SARS-CoV-2, where sterilizing immunity is difficult to achieve due to rapid antigenic evolution (Wahl and Wardemann, 2022). In this context, T-cell responses play a crucial role in mitigating disease severity by targeting conserved viral epitopes, thereby ensuring long-term protection even when neutralizing antibodies wane (Sette and Crotty, 2021). Unlike antibodies, which are more susceptible to immune escape, T cells recognize infected cells and contribute to viral clearance, reducing the risk of severe outcomes.

Concerning VBDs, the TBVs aim to disrupt pathogen development within the vector, thereby preventing transmission to the vertebrate host. TBVs typically involve immunizing vertebrate hosts with molecules derived either from the pathogen or the vector to hinder or reduce pathogen transmission to uninfected hosts, moving beyond the traditional pathogen-centered vaccine model (Coutinho-Abreu and Ramalho-Ortigao, 2010; Neelakanta and Sultana, 2015). In malaria, TBV development has been primarily focused on antigens expressed on the surface of gametes, zygotes, and ookinetes. For example, the N-terminal pro domain of Pfs230, an antigen expressed on male and female gametes, was sufficient to induce complement-dependent transmission-blocking activity against *P. falciparum*, resulting in a significant reduction in oocyst numbers (Tachibana et al., 2011). Accordingly, Pfs230, Pfs48/45 and Pfs25 are the three leading TBV candidates that target certain proteins expressed on the sexual stages of *Plasmodium* within the mosquito, effectively blocking the infectivity and spread of the malarial parasite (Duffy, 2021; Healy et al., 2021; Sagara et al., 2023). Beyond malaria, TBV strategies are being developed for other VBDs, including Lyme disease caused by *B. burgdorferi*. The candidate vaccine VLA15, which includes OspA variants from six different serotypes, has undergone phase 1 clinical trials demonstrating safety, tolerability, and robust immunogenicity across all targeted serotypes (Bézay et al., 2023). These findings support the potential of antigen-specific antibodies to block transmission by interfering with the biological function of key pathogen proteins, thereby disrupting the cycle of infection.

Vector-targeted vaccines are a type of TBVs aimed at targeting vector molecules to block pathogen transmission from arthropods to mammalian hosts (Neelakanta and Sultana, 2015). These vaccines hold the potential to prevent the transmission of a variety of VBPs by using a single vaccine. Since vector saliva plays a crucial role in facilitating pathogen transmission and modulating host immune responses, often contributing to disease progression, several salivary proteins have been identified and proposed as promising vaccine candidates (Olajiga et al., 2021).

For instance, vaccination with PdSP15, a salivary protein from a sand fly, significantly reduced parasite burden after cutaneous leishmaniasis challenge in non-human primates (Oliveira et al., 2015). In mosquitoes, a synthetic peptide vaccine known as AGS-v, targets *Anopheles gambiae* saliva proteins. AGS-v comprises four conserved peptides predicted to be T-cell epitopes shared among *Anopheles*, *Aedes*, and *Culex* mosquitoes. In a phase 1 clinical trial, the vaccine was well tolerated and, when adjuvanted, induced robust immune responses, including elevated IgG and IFN-γ levels (Manning et al., 2020). In addition to salivary antigens, proteins from other vector organs have also been investigated as potential vaccine targets. Anti-tick vaccines represent a successful example of this strategy, particularly in livestock (de la Fuente et al., 2007; Rodríguez-Mallon, 2023). For instance, vaccines targeting the Bm86 protein, which is expressed in the midgut of the tick *R. microplus*, have effectively inhibited tick feeding and reduced pathogen transmission in cattle (de la Fuente et al., 2007).

Although recombinant protein-based vaccines have served as the cornerstone of vaccinology for the past century, offering safe, scalable, and well-characterized approaches to immunization, rapid advances in molecular biology, immunology, and biotechnology over the last three decades have highlighted the potential of alternative vaccine platforms. This includes the mRNA-based vaccines, which enable real-time updates and rapid vaccine redesigns to address antigenic drift and escape mutations, partially acknowledging the limitations of durable immune memory. These vaccines simplify the production process by directly synthesizing mRNA, which host cells use to produce antigens that are immediately presented to the immune system, eliciting both B and T cell-mediated responses essential for achieving effective and long-lasting immunity (Pardi et al., 2018). This approach has been successfully implemented in SARS-CoV-2 mRNA vaccines tailored to emerging variants and has potential applications in influenza and other rapidly evolving pathogens (Corbett et al., 2020; Chivukula et al., 2021).

Regarding vector-borne viruses, various mRNA vaccines targeting genes encoding viral surface proteins have been tested to date, including those for the E protein of DENV, ZIKV, Japanese encephalitis virus (JEV), CHIKV, and yellow fever virus (YFV), as well as the Gn and Gc glycoproteins of Rift Valley fever virus (RVFV) (Samsa et al., 2019; Zhong et al., 2019; Zhang et al., 2020; August et al., 2021; Chen et al., 2022; Khan et al., 2023; Sun et al., 2025). Mice immunized with an mRNA vaccine containing envelope domain III (E-DIII) and non-structural protein 1 (NS1), encapsulated in lipid nanoparticles, developed a robust antiviral immune response and increased neutralizing antibody titers that effectively blocked all four DENV serotypes *in vitro* without significant ADE (He et al., 2022). Moreover, a comparison of immunogenicity and protective efficacy between an OspA-encoding mRNA-LNP vaccine and an alum-adjuvanted OspA protein subunit vaccine targeting *B. burgdorferi* showed that the mRNA-based vaccine induced superior humoral and cell-mediated immune responses in mice after a single immunization (Pine et al., 2023). Together, these studies demonstrate mRNA-based vaccines as a promising strategy for developing effective vaccines against viral and bacterial VBPs.

mRNA vaccines have also been used to target vector salivary proteins. For example, a vaccine based on lipid nanoparticles containing an mRNA encoding AgTRIO, a mosquito saliva protein, has been shown to reduce *Plasmodium* liver infection and improve survival in mice (Chuang et al., 2023). Moreover, an mRNA vaccine encoding 19 *I. scapularis* salivary proteins (19ISP) elicited robust humoral and cellular immune responses, partially inhibited tick feeding, and caused premature detachment, thereby reducing pathogen transmission (Sajid et al., 2021). This combination of multi-antigenic vaccines with mRNA platforms represents a promising strategy for developing vaccines aimed at reducing the transmission of VBPs.

Furthermore, anti-microbiota vaccines have emerged as an innovative strategy to target the vector’s microbiota rather than the pathogen itself, disrupting vector physiology or pathogen colonization by manipulating the internal microbial ecology of the vector (Wu-Chuang et al., 2023). These vaccines can induce host antibodies against specific microbial taxa within the vector’s microbiota (Mateos-Hernández et al., 2021; Cano-Argüelles et al., 2024). Once ingested by the vector during feeding, these antibodies can target symbiotic bacteria that support pathogen survival or transmission. For instance, anti-microbiota vaccine targeting keystone bacteria in the tick gut reduced *Borrelia afzelii* colonization (Wu-Chuang et al., 2023). Similarly, anti-microbiota vaccine induced avian host antibodies that manipulated mosquito microbiota and reduced *Plasmodium* development in the mosquito midgut (Aželytė et al., 2022). Anti-microbiota vaccine could also be designed to target symbiotic bacteria for reducing vector fitness, which ultimately will reduce pathogen circulation. This approach may complement traditional TBVs by interfering with pathogen colonization at early stages. However, this method requires a deep understanding microbiota-pathogen-vector interactions, a field still in its early stages, and faces challenges in achieving sustained microbiota dysbiosis.

Overall, new approaches for vaccine development, such as transmission-blocking and anti-microbiota vaccines, offers the possibility of community-level transmission control, shifting the focus from individual protection – as emphasized in classical vaccines developed under the Jenner/Pasteur paradigm – to population-wide disease mitigation. However, to be effective, their implementation may need to be integrated with complementary strategies such as environmental management and vector control to achieve successful and sustained control of VBDs.

## 7 Concluding remarks

Vaccination, guided by the Jenner/Pasteur paradigm, has transformed public health, achieving remarkable successes in controlling many infectious diseases. However, as pathogens continue to adapt, evade, and persist, no single approach can universally address the complexities of pathogen ecology. This review has highlighted how vaccination campaigns act as large-scale ecological experiments, revealing the diverse strategies VBPs use to survive under immune pressure and exposing the limits of traditional vaccine approaches.

The insights gained from these efforts underscore the need for a broader, more integrated view of vaccination. While the Jenner/Pasteur paradigm remains a powerful foundation, are there untapped vaccine strategies, such as targeting host-pathogen interactions or vector-specific components, that could circumvent resistance mechanisms revealed by the paradigm? What determines a pathogen’s susceptibility or resistance to vaccine-induced immunity across diverse ecological contexts? How can real-time genomic surveillance be integrated with vaccine development to address rapidly evolving pathogens like dengue? How can we predict which pathogens are most likely to evolve antigenic variation in response to widespread vaccination efforts? Future strategies must incorporate ecological, evolutionary, and immunological perspectives to address the challenges posed by pathogens resistant to vaccine-induced immunity. Key areas for progress include the development of vaccines targeting conserved antigens, leveraging real-time genomic surveillance to adapt to antigenic variation, and combining vaccination with ecological interventions, such as vector control or environmental sanitation.

Importantly, VBPs that escape vaccine-induced immunity remind us of the dynamic nature of the host-pathogen relationship. As vaccines shape the evolutionary trajectories of pathogens, new questions arise about the long-term impacts of vaccination on pathogen diversity, transmission dynamics, and population-level immunity. For instance, what ecological conditions favor latency or chronic infection as a persistence strategy, and how can vaccines or adjunct therapies disrupt these mechanisms? To what extent do environmental factors (e.g. climate, urbanization, habitat changes) influence the success of vaccination campaigns against pathogens with alternative survival strategies? What lessons from pathogens that escape vaccine-induced immunity can guide future public health approaches to emerging infectious diseases? Addressing these questions will require interdisciplinary collaboration across immunology, ecology, and public health.

Looking forward, vaccination strategies must be as adaptable and innovative as the pathogens they target. By embracing the complexity of pathogen ecology and integrating cutting-edge scientific advances, we can design interventions that not only respond to current challenges but also anticipate future ones. In doing so, we continue the legacy of the Jenner/Pasteur paradigm while expanding its scope to meet the demands of an ever-evolving infectious disease landscape.

## CRediT authorship contribution statement

**Ana Laura Cano-Argüelles:** Conceptualization, Writing – original draft, Writing – review & editing, Visualization. **Lianet Abuin-Denis:** Writing – review & editing. **Dasiel Obregon:** Visualization, Writing – review & editing. **Lourdes Mateos-Hernandez:** Writing – review & editing. **Apolline Maître:** Writing – review & editing. **Elianne Piloto-Sardiñas:** Writing – review & editing. **Alejandra Wu-Chuang:** Writing – review & editing. **Pierre Tonnerre:** Conceptualization, Supervision, Writing – original draft, Writing – review & editing. **Alejandro Cabezas-Cruz:** Conceptualization, Supervision, Writing – original draft, Writing – review & editing.

## Ethical approval

Not applicable.

## Funding

Alejandro Cabezas-Cruz is supported by the French Government’s Investissement d’Avenir program, Laboratoire d’Excellence “Integrative Biology of Emerging Infectious Diseases” (grant no. ANR-10-LABX-62-IBEID). Pierre Tonnerre is supported by the ATIP-Avenir program from INSERM and CNRS. Ana Laura Cano-Argüelles is hired under the Generation D initiative, promoted by Red.es, an organisation attached to the Ministry for Digital Transformation and the Civil Service, for the attraction and retention of talent through grants and training contracts, financed by the Recovery, Transformation and Resilience Plan through the European Union’s Next Generation funds.

## Declaration of competing interests

The authors declare that they have no known competing financial interests or personal relationships that could have appeared to influence the work reported in this paper. Given their role as Guest Editor, Alejandro Cabezas-Cruz had no involvement in the peer review of this article and has no access to information regarding its peer review. Full responsibility for the editorial process for this article was delegated to Dr Frank Katzer (Co-Editor) and Professor Aneta Kostadinova (Editor-in-Chief).

## Data Availability

The data supporting the conclusions of this article are included within the article.
